# African Swine Fever Virus pI215L Inhibits Type I Interferon Signaling by Targeting Interferon Regulatory Factor 9 for Autophagic Degradation

**DOI:** 10.1128/jvi.00944-22

**Published:** 2022-08-16

**Authors:** Liang Li, Jiyang Fu, Jixuan Li, Shibang Guo, Qichao Chen, Yibo Zhang, Zhankui Liu, Chen Tan, Huanchun Chen, Xiangru Wang

**Affiliations:** a State Key Laboratory of Agricultural Microbiology, College of Veterinary Medicine, Huazhong Agricultural University, Wuhan, China; b Key Laboratory of Preventive Veterinary Medicine in Hubei Province, The Cooperative Innovation Center for Sustainable Pig Production, Wuhan, China; c Key Laboratory of Prevention & Control for African Swine Fever and Other Major Pig Diseases, Ministry of Agriculture and Rural Affairs, Wuhan, China; d International Research Center for Animal Disease, Ministry of Science and Technology of the People’s Republic of China, Wuhan, China; Hudson Institute of Medical Research

**Keywords:** African swine fever virus, pI215L, type I IFN signaling, IRF9, autophagy

## Abstract

African swine fever virus (ASFV) is the etiological agent of a highly lethal hemorrhagic disease in domestic pigs and wild boars that has significant economic consequences for the pig industry. The type I interferon (IFN) signaling pathway is a pivotal component of the innate antiviral response, and ASFV has evolved multiple mechanisms to antagonize this pathway and facilitate infection. Here, we reported a novel function of ASFV pI215L in inhibiting type I IFN signaling. Our results showed that ASFV pI215L inhibited IFN-stimulated response element (ISRE) promoter activity and subsequent transcription of IFN-stimulated genes (ISGs) by triggering interferon regulatory factor 9 (IRF9) degradation. Additionally, we found that catalytically inactive pI215L mutations retained the ability to block type I IFN signaling, indicating that this only known viral E2 ubiquitin-conjugating enzyme mediates IFR9 degradation in a ubiquitin-conjugating activity-independent manner. By coimmunoprecipitation, confocal immunofluorescence, and subcellular fractionation approaches, we demonstrated that pI215L interacted with IRF9 and impaired the formation and nuclear translocation of IFN-stimulated gene factor 3 (ISGF3). Moreover, further mechanism studies supported that pI215L induced IRF9 degradation through the autophagy-lysosome pathway in both pI215L-overexpressed and ASFV-infected cells. These findings reveal a new immune evasion strategy evolved by ASFV in which pI215L acts to degrade host IRF9 via the autophagic pathway, thus inhibiting the type I IFN signaling and counteracting the host innate immune response.

**IMPORTANCE** African swine fever virus (ASFV) causes a highly contagious and lethal disease in pigs and wild boars that is currently present in many countries, severely affecting the global pig industry. Despite extensive research, effective vaccines and antiviral strategies are still lacking, and many fundamental questions regarding the molecular mechanisms underlying host innate immunity escape remain unclear. In this study, we identified ASFV pI215L, the only known viral E2 ubiquitin-conjugating enzyme, which is involved in antagonizing the type I interferon signaling. Mechanistically, pI215L interacted with interferon regulatory factor 9 for autophagic degradation, and this degradation was independent of its ubiquitin-conjugating activity. These results increase the current knowledge regarding ASFV evasion of innate immunity, which may instruct future research on antiviral strategies and dissection of ASFV pathogenesis.

## INTRODUCTION

African swine fever (ASF) is an acute hemorrhagic and highly contagious disease in domestic pigs and wild boars caused by African swine fever virus (ASFV) (1, 2). Since its first identification in Kenya in 1921 (3), ASF has been distributed in most sub-Saharan African countries, the Russian Federation, TransCaucasus, some Eastern and Central European countries, Sardinia, and Southeast and East Asia, seriously threatening the global pig industry and food security (4–7). Given the threat the disease poses to global agriculture and trade, ASF is listed as a notifiable disease by the World Organization for Animal Health (OIE) (8–10). Unfortunately, there are no approved commercial vaccines or treatments available for ASF, and control of the disease depends on the implementation of rigorous import policies and biosecurity measures with costly socioeconomic impacts (11, 12). The recent ASF pandemics in China and neighboring countries in Asia have caused an estimated direct economic loss of $55 to $130 billion (13).

ASFV is the only characterized member of the *Asfarviridae* family and the only known DNA arbovirus (14, 15). It has a large linear double-stranded DNA genome of approximately 170 to 194 kbp containing more than 150 open reading frames (ORFs), with half of them lacking any known or predictable function (16–18). ASFV predominantly replicates in pig monocytes and macrophages (19). Since these cells play critical roles in activating and orchestrating the host innate and adaptive immune responses, ASFV has evolved numerous strategies to evade immune defenses through a highly coordinated process that depends on the temporally and spatially regulated expression of different viral gene categories (20–23). The giant genome and complex immune escape mechanisms pose challenges to ASFV immune prevention and vaccine development (4, 24).

As the first line of defense against viral infection, type I interferons (IFNs) play a pivotal role in the innate immune response (25, 26). Type I IFN production is initiated upon recognition of pathogen-associated molecular patterns (PAMPs) by host pattern recognition receptors (PRRs) (27). These receptors trigger the transduction of signaling cascades, leading to the secretion of type I IFN (27). Subsequently, type I IFNs bind to their surface receptors, IFNAR1 and IFNAR2, and activate the phosphorylation of Janus kinase 1 (JAK1) and tyrosine kinase 2 (TYK2) (28). Activated JAK1 and TYK2 phosphorylate signal transducer and activator of transcription 1 (STAT1) and STAT2, followed by interaction with interferon regulatory factor 9 (IRF9) to form a heterotrimer termed IFN-stimulated gene factor 3 (ISGF3) (29, 30). ISGF3 translocates into the nucleus and binds to the IFN-stimulated response element (ISRE), resulting in the activation of IFN-stimulated gene (ISG) transcription, which contributes to the establishment of the antiviral state in the cells (29, 31).

It is well known that viruses have developed multiple strategies to evade cellular antiviral defenses and modulate gene expression, thereby initiating a productive infection, such as encoding ubiquitin-related enzymes to subvert the ubiquitin-proteasome system of host cells (32–35). Interestingly, ASFV encodes the only known viral E2 ubiquitin-conjugating enzyme (pI215L) that shares a 30 to 48% amino acid identity with its eukaryotic counterparts (36, 37). A previous study revealed that pI215L dynamically shuttles between the nucleus and cytoplasm and changes along with infection (37). pI215L is expressed as an early protein and plays a critical role in the transcription of late viral genes and viral DNA replication (38). Furthermore, as previously shown, pI215L was able to regulate host protein translation by hijacking cellular components that impact the mTORC signaling pathway (37). Recently, it has been reported that ASFV pI215L was one of the strongest inhibitors in modulating the type I IFN production by antagonizing the cGAS-STING pathway; knockdown of pI215L expression enhanced type I IFN production and inhibited ASFV replication (39). However, whether pI215L is involved in blocking type I IFN signaling cascade and the underlying mechanisms remains unclear.

In this study, we demonstrated that ASFV pI215L substantially reduced the expression of IRF9, a key molecule in the ISGF3 complex, thereby inhibiting the type I IFN signaling pathway in a ubiquitin-conjugating activity-independent manner. More importantly, we showed that pI215L specifically interacted with IRF9 for its degradation through an autophagy-lysosome-dependent mechanism. Our results reveal a novel function of ASFV pI215L in type I IFN signaling and a previously unidentified strategy employed by ASFV to escape host innate immunity.

## RESULTS

### Identification of ASFV pI215L as an antagonist of type I IFN signaling.

**FIG 1:**
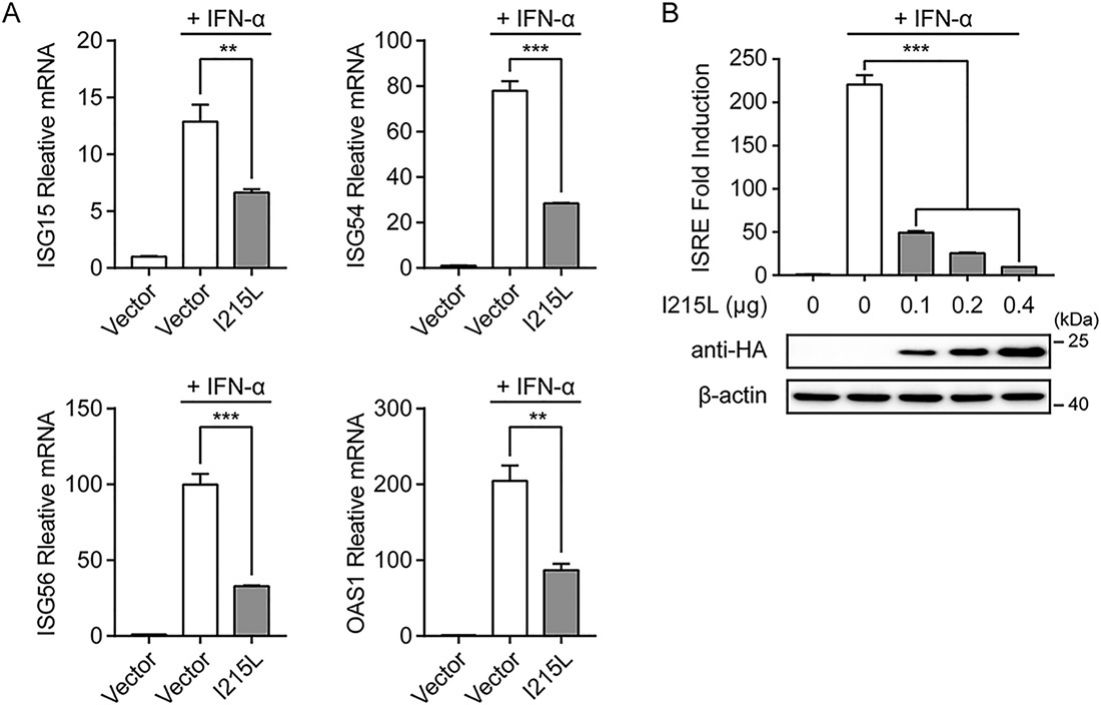
ASFV pI215L antagonizes type I IFN signaling. (A) HEK-293T cells cultured in 6-well plates were transfected with HA-tagged ASFV pI215L or empty vector. After 24 h, cells were treated with IFN-α (1,000 U/mL) for 8 h. The mRNA levels of ISG15, ISG54, ISG56, and OAS1 were analyzed by qRT-PCR. Data are representative of three independent experiments with *n *= 3 technical replicates (shown as mean ± SEM). (B) HEK-293T cells were seeded in 24-well plates and cotransfected with various concentrations of HA-tagged ASFV pI215L along with pISRE-Luc and pRL-TK plasmids. After 24 h, cells were treated with IFN-α (1,000 U/mL) for 12 h, followed by luciferase assays. The expression levels of pI215L were evaluated using immunoblotting analysis. Data are representative of three independent experiments with *n *= 3 technical replicates (shown as mean ± SEM). **, *P < *0.01; ***, *P < *0.001.

Type I IFN signaling induces a potent antiviral response in cells by inducing the expression of hundreds of IFN-stimulated genes (ISGs), which is vital for controlling viral infections (40). To assess the potential role of ASFV pI215L in type I IFN signaling, the mRNA levels of IFN-stimulated gene 15 (ISG15), ISG54, ISG56, and 2′-5′-oligoadenylate synthetase 1 (OAS1) were analyzed in human embryonic kidney cells (HEK-293T) overexpressing hemagglutinin (HA)-tagged ASFV pI215L. As shown in Fig. 1A, ASFV pI215L significantly inhibited the IFN-α-induced transcription of ISGs compared with the empty vector. Owing to the presence of the IFN-stimulated response element (ISRE) in the ISG promoter regions (28), HEK-293T cells were cotransfected with various concentrations of ASFV pI215L expression plasmid, along with the ISRE-luciferase and *Renilla* luciferase reporter plasmids. The results showed that pI215L strongly attenuated IFN-α-induced ISRE promoter activity in a dose-dependent manner in HEK-293T cells (Fig. 1B). These results confirm the antagonistic character of ASFV pI215L in type I IFN signaling.

### ASFV pI215L decreases IRF9 at the protein level.

**FIG 2:**
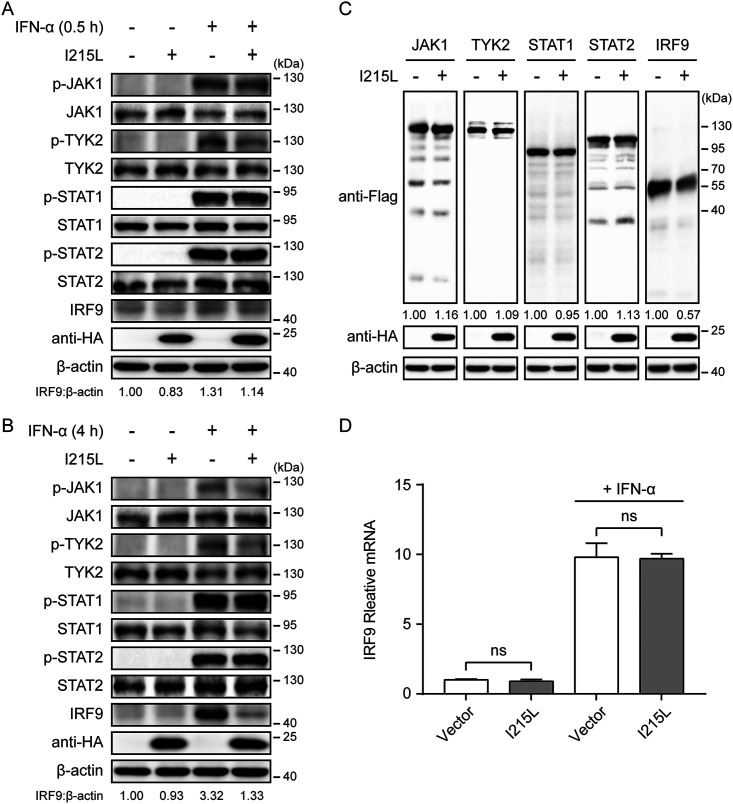
ASFV pI215L inhibits type I IFN signaling by decreasing IRF9 at the protein level. (A and B) HEK-293T cells were transfected with HA-tagged ASFV pI215L or empty vector. After 24 h, cells were treated with IFN-α (1,000 U/mL) for 0.5 h (A) or 4 h (B) and collected for immunoblotting analysis. Antibodies against JAK1, TYK2, STAT1, STAT2, IRF9, phospho-JAK1 (p-JAK1), phospho-TYK2 (p-TYK2), phospho-STAT1 (p-STAT1), and phospho-STAT2 (p-STAT2) were utilized to determine each respective endogenous protein. (C) HEK-293T cells were cotransfected with Flag-tagged porcine JAK1, TYK2, STAT1, STAT2, or IRF9 along with HA-tagged ASFV pI215L or empty vector. Cells were lysed at 30 h posttransfection and assessed by immunoblotting analysis. (D) HEK-293T cells in 6-well plates were transfected with HA-tagged ASFV pI215L or empty vector. After 24 h, cells were treated with IFN-α (1,000 U/mL) for 8 h. The mRNA level of IRF9 was measured by qRT-PCR. Data are representative of three independent experiments with *n *= 3 technical replicates (shown as mean ± SEM). ns, not significant (*P > *0.05).

To investigate the mechanism by which ASFV pI215L inhibits type I IFN signaling, HEK-293T cells were transfected with ASFV pI215L expression plasmid, and the endogenous protein levels and phosphorylation of crucial adaptor molecules in the type I IFN signaling pathway were examined in the presence or absence of IFN-α. The expression and phosphorylation of JAK1, TYK2, STAT1, and STAT2 were unaffected by ASFV pI215L (Fig. 2A and B). A slight reduction in IRF9 protein level was observed after IFN-α treatment for 0.5 h (Fig. 2A). However, the expression of IRF9 was significantly reduced by ASFV pI215L after IFN-α treatment for 4 h (Fig. 2B). To further elucidate the mechanism underlying the depletion of IRF9 mediated by ASFV pI215L, HEK-293T cells were cotransfected with pI215L along with Flag-tagged porcine JAK1, TYK2, STAT1, STAT2, or IRF9. Consistent with the above-described results, porcine IRF9 was markedly downregulated by ASFV pI215L (Fig. 2C). Since ASFV pI215L degrades IRF9 at the protein level, we next evaluated whether pI215L affects IRF9 expression at the transcriptional level with or without IFN-α stimulation. The results indicated that IRF9 mRNA levels were unaltered following transfection with ASFV pI215L (Fig. 2D). Together, these data demonstrate that ASFV pI215L inhibits type I IFN signaling by targeting IRF9 for degradation.

### ASFV pI215L induces IRF9 degradation in a ubiquitin-conjugating activity-independent manner.

**FIG 3:**
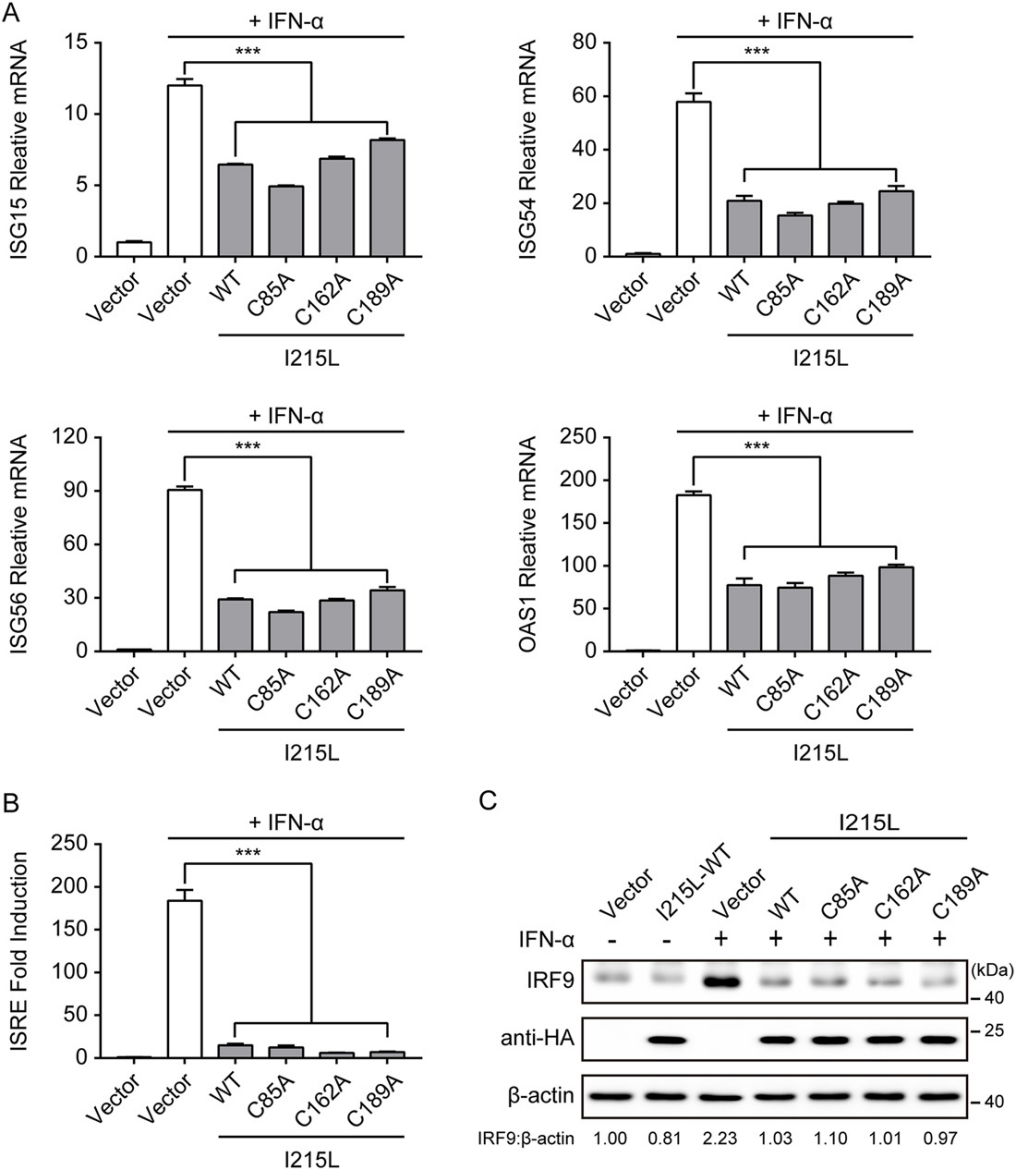
ASFV pI215L-mediated degradation of IRF9 is independent of its ubiquitin-conjugating activity. (A) HEK-293T cells cultured in 6-well plates were transfected with empty vector or HA-tagged ASFV wild-type (WT) pI215L or HA-tagged pI215L ubiquitin-conjugating activity defective mutation (C85A, C162A, or C189A). After 24 h, cells were treated with IFN-α (1,000 U/mL) for 8 h. The mRNA levels of ISG15, ISG54, ISG56, and OAS1 were analyzed by qRT-PCR. Data are representative of three independent experiments with *n *= 3 technical replicates (shown as mean ± SEM). (B) HEK-293T cells in 24-well plates were cotransfected with HA-tagged ASFV pI215L or its mutations along with pISRE-Luc and pRL-TK plasmids. After 24 h, cells were treated with IFN-α (1,000 U/mL) for 12 h, followed by luciferase assays. Data are representative of three independent experiments with *n *= 3 technical replicates (shown as mean ± SEM). (C) HEK-293T cells were transfected with HA-tagged ASFV I215L or its mutations. After 24 h, cells were treated with IFN-α (1,000 U/mL) for 4 h and collected for immunoblotting analysis. The expression level of the endogenous IRF9 was determined using an anti-IRF9 antibody. ***, *P < *0.001.

Previous reports have shown that ASFV pI215L acts as an E2 ubiquitin-conjugating enzyme, and Cys85 residue plays an essential role in the transesterification reaction (37, 38). Therefore, ASFV pI215L may participate in hijacking the cellular ubiquitin-proteasome system, modulating the function and subcellular localization of host proteins, resulting in the ability of viruses to evade the host antiviral response by targeting proteins for proteasomal degradation (38). To evaluate whether the ubiquitin-conjugating activity of pI215L was involved in the inhibition of the type I IFN signaling pathway, three putative catalytic residue single-point mutations, C85A, C162A, and C189A, were introduced into pI215L. However, none of the mutations showed a loss of the ability to inhibit the IFN-α-induced transcription of ISGs in HEK-293T cells overexpressing HA-tagged pI215L mutations (Fig. 3A). Therefore, we further tested the ability of pI215L mutations to inhibit IFN-α-induced ISRE promoter activity. As shown in Fig. 3B, each pI215L mutation (C85A, C162A, or C189A) also inhibited IFN-α-induced ISRE promoter activity. In addition, similar to the results seen with wild-type pI215L, each mutation also significantly caused the degradation of IFN-α-induced IRF9 (Fig. 3C). These results strongly indicate that ASFV pI215L-mediated inhibition of type I IFN signaling via IRF9 degradation is independent of its ubiquitin-conjugating activity.

### ASFV pI215L mediates IRF9 degradation through an autophagy-lysosome pathway.

**FIG 4:**
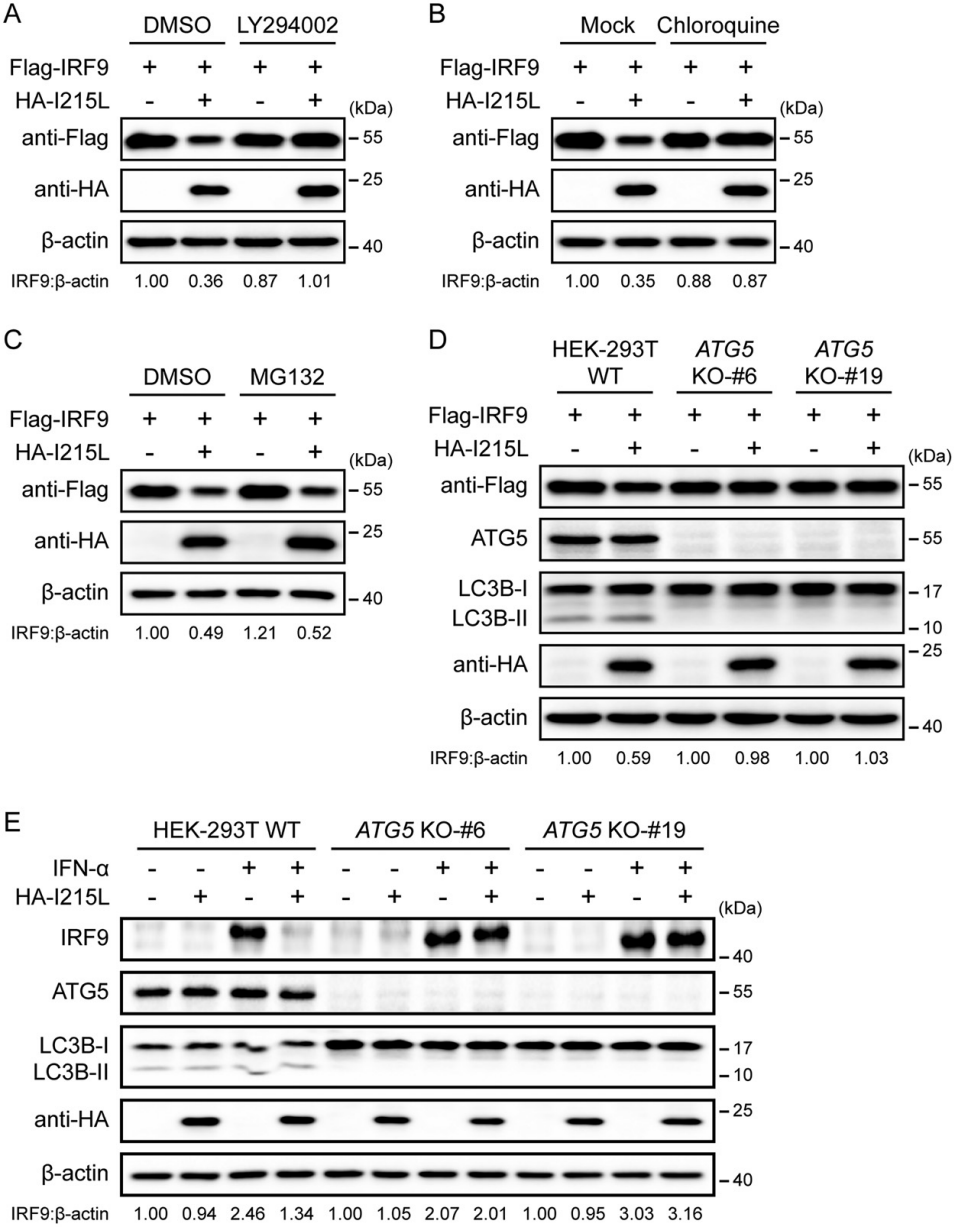
ASFV pI215L triggers autophagy-lysosome degradation of IRF9. (A to C) HEK-293T cells were cotransfected with Flag-tagged porcine IRF9 along with HA-tagged ASFV pI215L or empty vector. After 24 h, cells were then treated with LY294002 (10 μM) (A), chloroquine (50 μM) (B), or MG132 (10 μM) (C) for 6 h. Cell lysates were used for immunoblotting analysis with the indicated antibodies. DMSO, dimethyl sulfoxide. (D) Wild-type (WT) and *ATG5* KO HEK-293T cells were cotransfected with Flag-tagged porcine IRF9 along with HA-tagged ASFV pI215L or empty vector. Cells were lysed at 30 h posttransfection and assessed by immunoblotting analysis. (E) WT and *ATG5* KO HEK-293T cells were transfected with HA-tagged ASFV pI215L or empty vector. After 24 h, cells were treated with IFN-α (1,000 U/mL) for 4 h and harvested for immunoblotting analysis.

The ubiquitin-proteasome and the autophagy-lysosome pathways are the two major protein degradation pathways in eukaryotic cells (41). To illustrate the pathways involved in pI215L-mediated IRF9 degradation, HEK-293T cells cotransfected with Flag-IRF9 and HA-I215L expression vectors were treated with specific inhibitors which block protein degradation via the two above-described pathways. Both the autophagy inhibitor LY294002 (Fig. 4A) and lysosome inhibitor chloroquine (Fig. 4B) effectively blocked the IRF9 degradation mediated by pI215L. However, treatment with the proteasome inhibitor MG132 could not rescue IRF9 expression in the presence of ASFV pI215L expression (Fig. 4C). To further corroborate the involvement of autophagy in the degradation of IRF9 mediated by ASFV pI215L, a series of autophagy-related 5 (*ATG5*) knockout (KO) HEK-293T cell lines were generated using CRISPR/Cas9 technology, since ATG5 is essential for autophagosome formation (42). Consistent with the inhibitor treatment assay, the degradation of exogenous and endogenous IRF9 by ASFV pI215L was abolished in *ATG5* KO cells compared with wild-type HEK-293T cells (Fig. 4D and E). Collectively, these data indicate that the autophagy-mediated lysosomal pathway is responsible for the IRF9 degradation by ASFV pI215L.

### ASFV pI215L interacts with IRF9.

**FIG 5:**
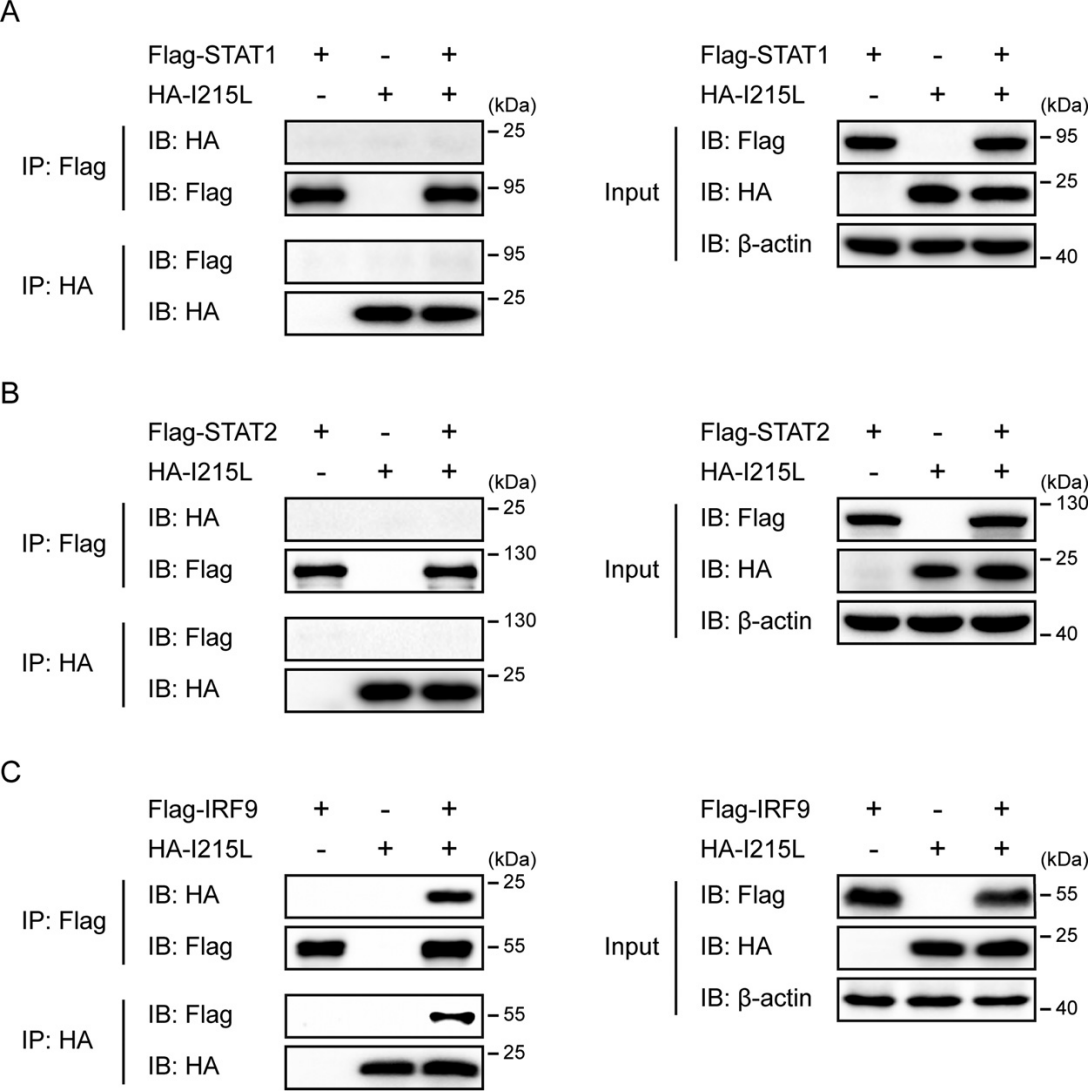
ASFV pI215L interacts with IRF9. (A to C) HEK-293T cells were cotransfected with Flag-tagged porcine STAT1 (A), STAT2 (B), or IRF9 (C) along with HA-tagged ASFV pI215L for 30 h. Cell lysates were immunoprecipitated using anti-Flag magnetic beads or anti-HA magnetic beads and subsequently analyzed by immunoblotting analysis with the indicated antibodies.

Previous studies have shown that several viral proteins inhibit type I IFN signaling by interacting with components of the IFN-stimulated gene factor 3 (ISGF3) complex (43–47). To investigate the possible interaction between ASFV pI215L and the components of ISGF3, HEK-293T cells were cotransfected with Flag-tagged porcine STAT1, STAT2, or IRF9 along with HA-tagged ASFV pI215L. The coimmunoprecipitation (co-IP) and immunoblotting analyses showed that ASFV pI215L was specifically coimmunoprecipitated with IRF9, but not STAT1 or STAT2, and the reverse co-IP experiment also confirmed the interaction between IRF9 and pI215L (Fig. 5A to C). These results revealed that the host IRF9 protein is a novel ASFV pI215L-interacting protein.

### ASFV pI215L impairs the IFN-α-stimulated formation and nuclear accumulation of ISGF3.

**FIG 6:**
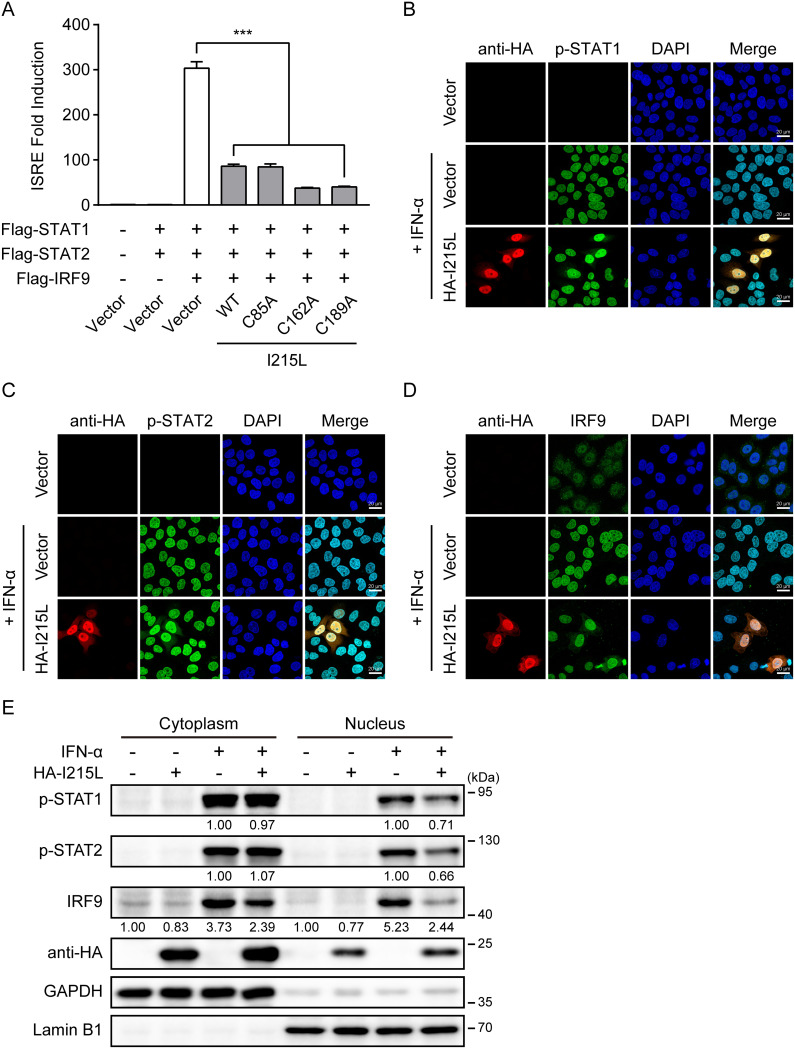
ASFV pI215L reduces the IFN-α-induced formation and nuclear accumulation of ISGF3. (A) HEK-293T cells were cotransfected with HA-tagged ASFV pI215L or its ubiquitin-conjugating activity-defective mutations (C85A, C162A, or C189A), along with Flag-tagged porcine ISGF3 complex (STAT1/STAT2/IRF9) and pISRE-Luc and pRL-TK plasmids. After 30 h, cells were harvested for luciferase assays. Data are representative of three independent experiments with *n *= 3 technical replicates (shown as mean ± SEM). ***, *P < *0.001. (B to D) HeLa cells were transfected with HA-tagged ASFV pI215L or empty vector. At 24 h posttransfection, cells were treated with IFN-α (1,000 U/mL) for 4 h. After the cells were fixed and permeabilized, they were incubated with the corresponding primary antibodies. Alexa Fluor 488-conjugated (green) secondary antibody was used to visualize endogenous p-STAT1 (B), p-STAT2 (C), or IRF9 (D) and Cy3-conjugated (red) secondary antibody to visualize pI215L. Nuclei (blue) were stained with DAPI. Scale bar, 20 μm. (E) HEK-293T cells were transfected with HA-tagged ASFV pI215L or empty vector. After 24 h, cells were treated with IFN-α (1,000 U/mL) for 4 h and harvested for subcellular fractionation. The nuclear and cytoplasmic fractions were subjected to immunoblotting analysis. As controls of the fractionation, nuclear antibody against lamin B1 and cytoplasmic antibody against GAPDH were used.

In the type I IFN-mediated signaling pathway, phosphorylated STAT1 and STAT2 heterodimerize and combine with IRF9 to form ISGF3, which translocates to the nucleus and activates ISRE promoter activity to generate a broad range of ISGs (29–31). Furthermore, high levels of unphosphorylated STAT1 and STAT2, as well as IRF9, contribute to the formation of unphosphorylated ISGF3, which activates ISRE and significantly increases the expression of ISGs (46–48). Given the pivotal role of ISGF3 in type I IFN signaling, we further investigated whether overexpression of pI215L inhibits ISGF3-mediated signaling. As shown in Fig. 6A, coexpression of the transcription factor complex ISGF3 components (STAT1, STAT2, and IRF9) significantly activated the ISRE promoter activity compared with the empty vector controls. However, activation of the ISRE promoter by ISGF3 was observably inhibited by the presence of ASFV pI215L (Fig. 6A). Consistently, each pI215L mutation (C85A, C162A, or C189A) was also able to suppress the ISGF3-mediated ISRE promoter activity (Fig. 6A), suggesting that the ubiquitin-conjugating activity of pI215L does not govern the ability of pI215L to block ISGF3-induced ISRE promoter activity. Previous studies have revealed that the function of ISGF3 depends on the selective interaction between phosphorylated STAT2 and the IRF-association domain of IRF9 (49, 50). The observed interaction between ASFV pI215L and IRF9 led us to speculate that this interaction may impair the recruitment of phosphorylated STAT2 by IRF9 and the subsequent nuclear accumulation of ISGF3. To test this hypothesis, confocal immunofluorescence analyses were performed to analyze the effect of ASFV pI215L on IFN-α-stimulated nuclear accumulation of ISGF3. As expected, IFN-α-stimulated phosphorylated STAT1, phosphorylated STAT2, and IRF9 nuclear translocation were partially inhibited in HeLa cells transfected with pI215L (Fig. 6B to D). In addition, nuclear and cytoplasmic fractionation assays supported that ASFV pI215L reduced the levels of phosphorylated STAT1, phosphorylated STAT2, and IRF9 in the nuclear fraction after IFN-α treatment (Fig. 6E). Together, these data indicate that ASFV pI215L impairs the formation and nuclear accumulation of ISGF3.

### ASFV infection degrades IRF9 through the interaction of pI215L with IRF9.

**FIG 7:**
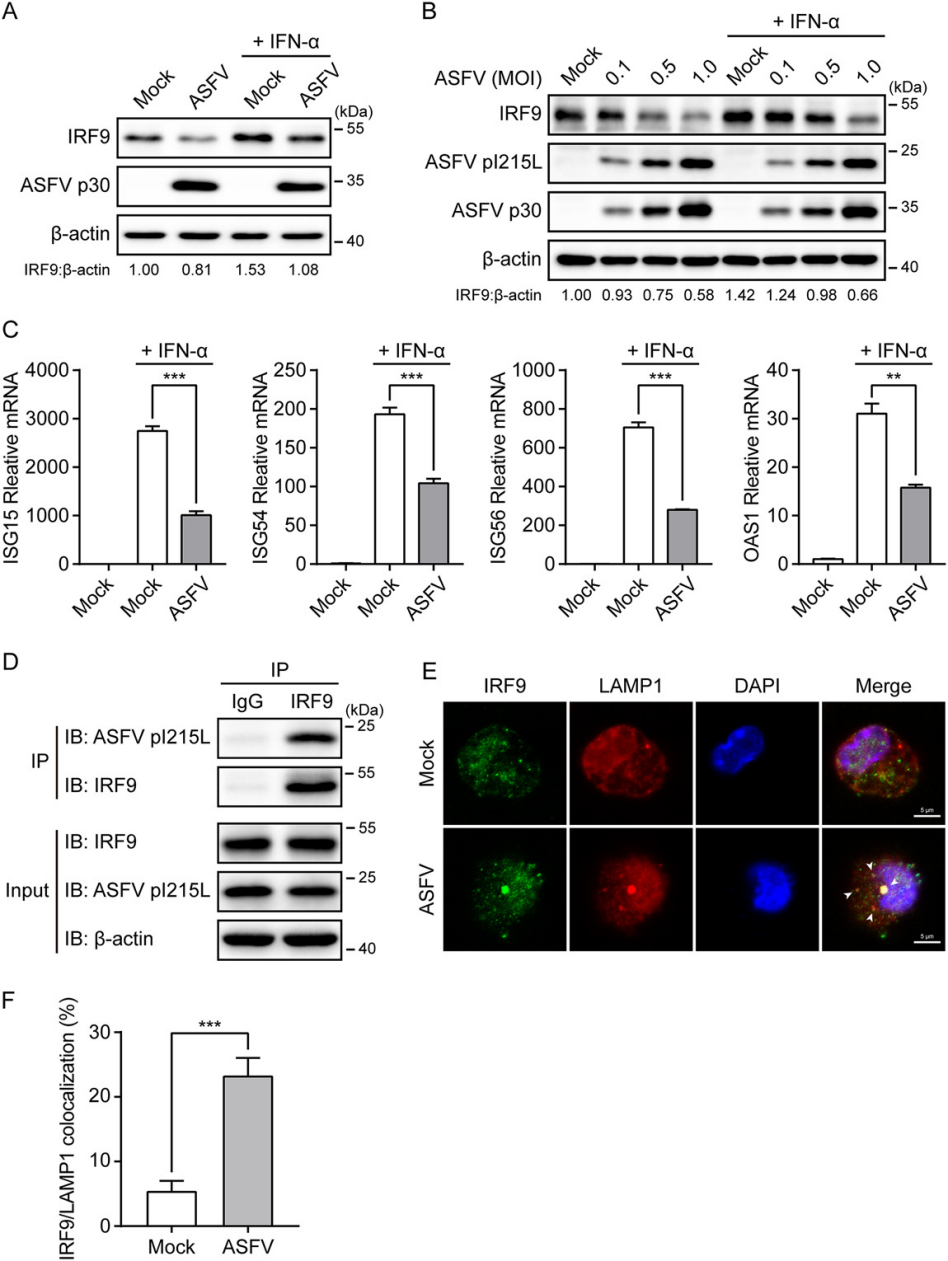
ASFV infection induces the autophagic degradation of IRF9 through the pI215L-IRF9 interaction. (A and B) PAM cells were infected with ASFV CN/SD/2019 at an MOI of 0.5 for 24 h (A) or were infected with ASFV at the indicated MOI for 48 h (B) in the presence or absence of the IFN-α (1,000 U/mL, 4 h prior to harvesting). The cell lysates were subjected to immunoblotting analysis. (C) PAM cells were infected with ASFV at an MOI of 0.5 for 24 h. Subsequently, cells were treated with IFN-α (1,000 U/mL) for 8 h. The mRNA levels of ISG15, ISG54, ISG56, and OAS1 were detected by qRT-PCR. Data are representative of three independent experiments with *n *= 3 technical replicates (shown as mean ± SEM). **, *P < *0.01; ***, *P < *0.001. (D) PAM cells were infected with ASFV at an MOI of 0.5 for 24 h. Cell lysates were immunoprecipitated with protein A/G magnetic beads precoated with anti-IRF9 antibody or rabbit IgG negative control and were then analyzed by immunoblotting analysis with the indicated antibodies. (E) PAM cells were infected with ASFV at an MOI of 0.5 for 24 h. After the cells were fixed and permeabilized, they were incubated with anti-IRF9 and anti-LAMP1 antibodies. Alexa Fluor 488- and Alexa Fluor 594-conjugated secondary antibodies were used to visualize IRF9 (green) and LAMP1 (red) proteins, respectively. Nuclei (blue) were stained with DAPI. The endogenous IRF9 colocalized with LAMP1 (lysosome marker) in the cytoplasm is indicated by white arrows (ASFV infection group). Scale bar, 5 μm. (F) Histogram showing the percentages of cells with IRF9/LAMP1 colocalization. Data are representative of three independent experiments of >100 cells per group (shown as mean ± SEM). ***, *P < *0.001.

We next sought to verify the expression changes of IRF9 during ASFV infection of primary porcine alveolar macrophage (PAM) cells. A significant endogenous IRF9 degradation was observed in PAM cells infected with ASFV at a multiplicity of infection (MOI) of 0.5 for 24 h in the presence or absence of IFN-α (Fig. 7A). Additionally, ASFV infection diminished IRF9 levels in PAM cells in a dose-dependent manner (Fig. 7B). The quantitative real-time PCR (qRT-PCR) results further illustrate that ASFV infection notably inhibited the IFN-α-induced transcription of ISGs (Fig. 7C). Moreover, we performed the co-IP assays in ASFV-infected PAM cells to confirm the interaction between endogenous IRF9 and ASFV pI215L. As shown, endogenous IRF9 coimmunoprecipitated with the pI215L in the ASFV-infected cells (Fig. 7D). Furthermore, confocal microscopy showed that IRF9 colocalized with the lysosome marker lysosomal-associated membrane protein 1 (LAMP1) upon ASFV infection (Fig. 7E and F), firmly supporting the involvement of the autophagy-lysosome pathway in the IRF9 degradation in ASFV-infected cells. These results demonstrate that ASFV infection triggers IRF9 autophagic degradation through pI215L-IRF9 interaction.

## DISCUSSION

ASFV has a tropism for monocytes and macrophages, which play critical roles in disease pathogenesis, viral persistence, and dissemination (6, 8). Growing evidence has shown that ASFV has developed various mechanisms to evade the host innate immune response. The type I IFN pathway was suppressed in macrophages infected with highly pathogenic ASFV (51, 52). According to previous reports, ASFV-encoded multigene families 360 (MGF360) and MGF505/530 play crucial roles in determining macrophage host range (53) and were associated with inhibition of the type I IFN response (21–24, 54). In addition, ASFV A238L explicitly inhibited tumor necrosis factor-alpha (TNF-α) transcription through a mechanism that involves CBP/p300 (55). At the same time, ASFV I329L blocked the Toll-like receptor 3 signaling pathway through a crucial intracellular signaling adaptor molecule TRIF (56), and A179L interacted with proapoptotic Bcl-2 family proteins in subverting premature host cell apoptosis (57). These findings indicate that modulation of the host innate immune response plays a vital role in the pathogenesis of ASFV. Thus, identifying the key genes and their corresponding proteins mediating such processes is of great significance for better understanding virus-host interactions and is fundamental for the rational design of effective ASFV vaccines. In this study, we characterized ASFV pI215L as a novel type I IFN signaling antagonist that binds and degrades the crucial adaptor molecule IRF9. Our observations reinforce the hypothesis that this viral E2 ubiquitin-conjugating enzyme plays a crucial role in ASFV evasion of host antiviral response, probably by controlling the ubiquitination status of the cellular proteins to proteasomal degradation and modulating the activity of viral proteins via different mechanisms (38).

It is well known that ubiquitylation is a posttranslational modification associated with various cellular processes (58). The fundamental contributors to this cascade are the ubiquitin-activating enzyme (E1), ubiquitin-conjugating enzyme (E2), and ubiquitin ligase enzyme (E3), which attach ubiquitin to the substrate (59). Previous studies have revealed that some viral proteins can interact with cellular E3 ubiquitin ligases and trigger their ubiquitylation, thereby establishing a productive infection (60–62). Recently, it has been demonstrated that SARS-CoV-2 ORF10 interacts with multiple members of a Cullin 2 (CUL2) RING E3 ligase complex that targets substrates for degradation (63). More importantly, herpesviruses and poxviruses also encode their E3 ligases to evade the host innate immune response and promote viral replication (64, 65). Interestingly, ASFV is exclusively the virus known to encode an E2 ubiquitin-conjugating enzyme, which is the product of ASFV gene *I215L* (36). ASFV pI215L has been implicated as having a possible role in modulating host gene transcription since it binds to a host ARID DNA-binding domain-containing protein SMCp, which is involved in transcription regulation (66). Moreover, pI215L interacts with the 40S ribosomal protein RPS23, the cap-dependent translation machinery initiation factor eIF4E, and the E3 ubiquitin ligase Cullin 4B, highlighting the relevance of this protein in regulating host protein translation (37). E2 ubiquitin-conjugating enzymes are central players in the enzymatic process of ubiquitylation, and previous studies have revealed the conjugating activity of pI215L, although the *in vivo* substrate for this viral enzyme has not been identified (38). In the present study, IRF9 protein was notably downregulated by ASFV pI215L. However, IRF9 mRNA levels were unaffected by transfection with pI215L, suggesting that pI215L might trigger the IRF9 polyubiquitination for the proteasome-dependent degradation. Unexpectedly, our results clearly showed that the catalytically inactive pI215L mutations (C85A, C162A, and C189A) retained the ability to disrupt type I IFN signaling by targeting IRF9 for degradation. Noticeably, recent studies have shown that ASFV pI215L negatively regulates the cGAS-STING signaling pathway and NF-κB signaling, both independent of its ubiquitin-conjugating activity (39, 67), which is consistent with our observation, suggesting that this multifunctional viral E2 ubiquitin-conjugating enzyme has evolved other strategies to manipulate the host innate immune response.

As a crucial component of the early host antiviral response, type I IFN signaling controls viral infection by activating the transcription factor complex ISGF3 (IRF9, STAT1, and STAT2), resulting in the coordinated upregulation of hundreds of ISGs that orchestrate an antiviral state in the cell (29, 31). It is becoming increasingly apparent that IRF9 is a central factor not only for mediating but also for regulating and directing the type I IFN response (68). Abundant evidence suggests that IRF9 is a common target hijacked by viral proteins. For example, porcine bocavirus (PBoV) nonstructural protein 1 (NS1) inhibited the DNA-binding activity of ISGF3 by interacting with IRF9 (45). Likewise, the nsp11 of the porcine reproductive and respiratory syndrome virus (PRRSV) bonded to IRF9 to suppress the formation and nuclear translocation of ISGF3 (46). Moreover, several virus-encoded proteins, such as adenovirus E1A, rotavirus NSP1, simian varicella virus (SVV) ORF63, and herpes simplex virus 2 (HSV-2) ICP22, mediated IRF9 degradation (69–72). In the current study, we showed that ASFV pI215L specifically interacted with IRF9 and induced the degradation of IRF9. More importantly, ASFV pI215L mediates IRF9 degradation through the autophagy-lysosome pathway, in contrast to the proteasome-dependent manner observed in SVV and HSV-2 induced IRF9 degradation (71, 72). To the best of our knowledge, these findings suggest a novel function of a viral E2 ubiquitin-conjugating enzyme that could degrade host proteins through autophagy.

Since pI215L is an essential viral protein for ASFV replication (37, 38, 73), we could not generate a defective viral mutant lacking the entire *I215L* gene to further assess the role of pI215L in IRF9 degradation in the context of viral infection. However, our results showed that endogenous IRF9 was coimmunoprecipitated with pI215L in the ASFV-infected cells, confirming the interaction between IRF9 and ASFV pI215L. In addition, we found that IRF9 colocalized with the lysosome upon ASFV infection, resulting in the degradation of IRF9 and reduced subsequent transcription of ISGs. These results indicated that ASFV could trigger IRF9 autophagic degradation through ASFV pI215L-IRF9 interaction, consistent with the observations in cells expressing pI215L in the transfection experiments.

**FIG 8:**
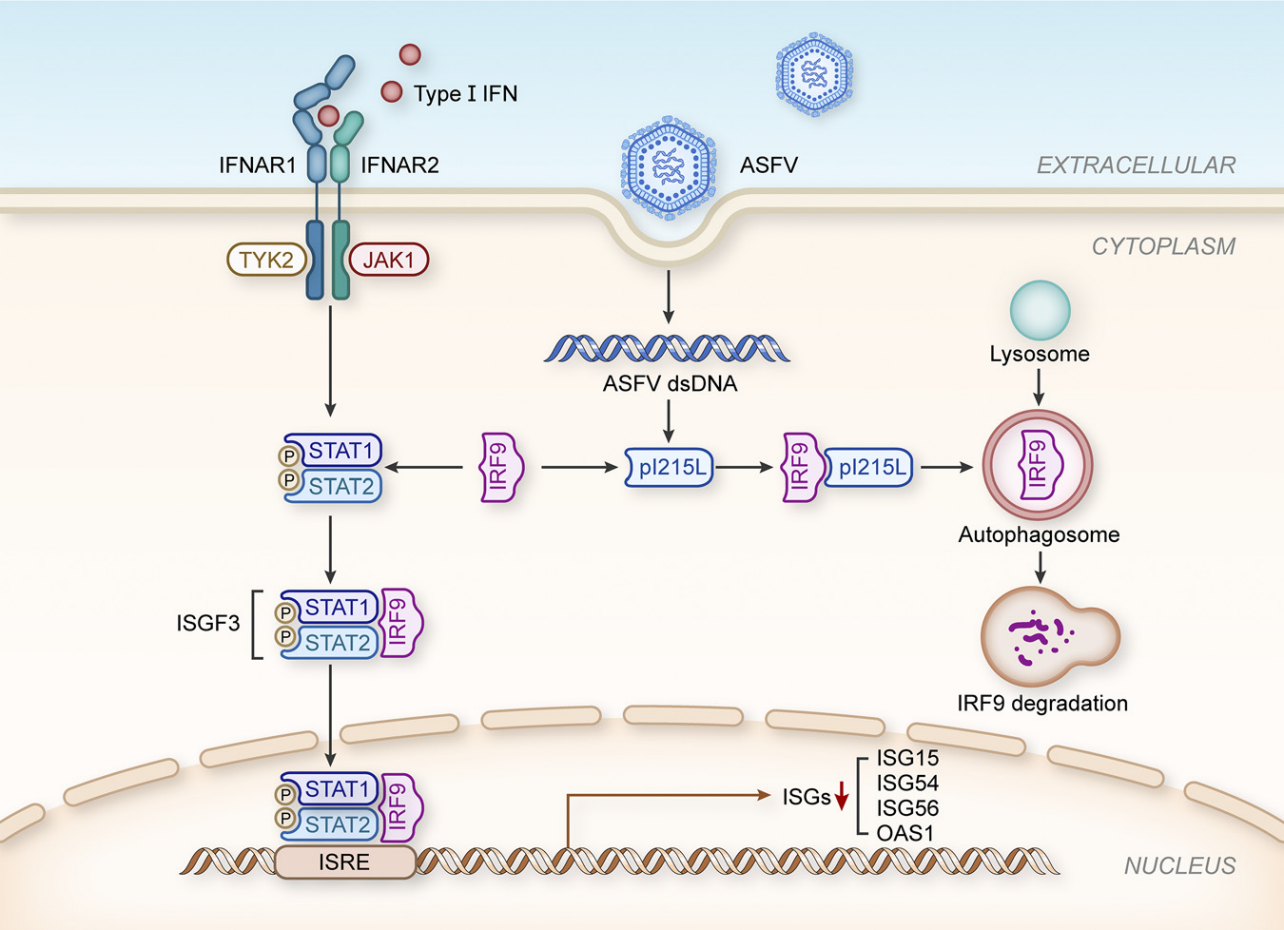
Schematic representation of the proposed role of ASFV pI215L in inhibiting type I IFN signaling. In ASFV-infected macrophages, ASFV-derived pI215L could interact with IRF9 and trigger IRF9 degradation through the autophagy-lysosome pathway to evade the host innate antiviral response.

In summary, our data reveal, for the first time, the use of autophagy by ASFV pI215L to degrade a type I IFN signaling factor independent of its ubiquitin-conjugating activity. These findings are schematically illustrated in the proposed molecular model of pI215L in Fig. 8. Although further work is primarily required to fully characterize how this viral protein achieves IRF9 degradation, this study highlights a new understanding regarding innate immune evasion mechanisms involving ASFV, which shall guide the future development of countermeasures against ASFV spreading globally.

## MATERIALS AND METHODS

### Cells and viruses.

HEK-293T (ATCC CRL-3216) and HeLa cells (ATCC CCL-2) were cultured in Dulbecco’s modified Eagle’s medium (DMEM) supplemented with 10% fetal bovine serum (FBS), 100 U/mL penicillin, and 100 μg/mL streptomycin. Primary porcine alveolar macrophage (PAM) cells were prepared by bronchoalveolar lavage as described previously (74) and cultured in RPMI 1640 medium supplemented with 10% FBS, 100 U/mL penicillin, and 100 μg/mL streptomycin. All cells were incubated at 37°C in a humidified atmosphere containing 5% CO_2_. The genotype II ASFV virulent isolate CN/SD/2019 was propagated and titrated using the hemadsorption (HAD) assay in PAM cells, as previously described (54).

### Plasmids.

Porcine JAK1, TYK2, STAT1, STAT2, and IRF9 were cloned into p3×Flag-CMV-14 with a C-terminal Flag tag. The ASFV gene *I215L* was amplified from ASFV CN/SD/2019 genomic DNA and cloned into pCAGGS-HA with an N-terminal HA tag. ASFV pI215L single-point mutations (C85A, C162A, and C189A) were generated by site-directed mutagenesis using the wild-type plasmid pCAGGS-HA-*I215L* as the template. All constructed plasmids were confirmed using DNA sequencing. The luciferase reporter plasmids pISRE-Luc and pRL-TK were kindly provided by Shaobo Xiao (Huazhong Agricultural University, Wuhan, China).

### Antibodies and reagents.

The JAK1 (3332S), phospho-JAK1 (74129S), TYK2 (9312S), phospho-TYK2 (9321S), STAT1 (9172S), phospho-STAT1 (7649S), STAT2 (4594S), phospho-STAT2 (88410S), IRF9 (76684S), and ATG5 (12994S) antibodies were purchased from Cell Signaling Technology (Danvers, MA, USA). β-actin (66009-1-Ig), lamin B1 (12987-1-AP), GAPDH (glyceraldehyde-3-phosphate dehydrogenase; 10494-1-AP), Flag-tag (20543-1-AP), and HA-tag (51064-2-AP, rabbit) antibodies were purchased from Proteintech (Chicago, IL, USA). LC3B (ab192890) and LAMP1 (ab25245) antibodies were purchased from Abcam (Cambridge, UK). The HA-tag antibody (AE008, mouse) was purchased from ABclonal (Wuhan, China). A polyclonal antibody against ASFV p30 was prepared in our laboratory. The secondary antibodies used for immunoblotting analysis, horseradish peroxidase (HRP)-conjugated goat anti-rabbit IgG (BF03008), and HRP-conjugated goat anti-mouse IgG (BF03001), were purchased from Biodragon (Beijing, China). The secondary antibodies used for immunofluorescence, included Alexa Fluor 488-conjugated goat anti-rabbit IgG (bs-0295G-AF488), Cy3-conjugated goat anti-mouse IgG (bs-0296G-Cy3), and Alexa Fluor 594-conjugated goat anti-rat IgG (bs-0293G-AF594) were purchased from Bioss (Beijing, China). Recombinant human IFN-α 2a (CYT-204) was purchased from ProSpec (Ness Ziona, Israel). The inhibitors MG132 (HY-13259), LY294002 (HY-10108), and chloroquine (HY-17589) were purchased from MedChemExpress (Monmouth Junction, NJ, USA). The jetPRIME transfection reagent was purchased from Polyplus-transfection SA (Illkirch, France).

### Dual-luciferase reporter assay.

HEK-293T cells were seeded in 24-well plates and transfected with the indicated expression plasmids or empty vector control, together with the firefly luciferase reporter plasmid pISRE-Luc (50 ng/well) and *Renilla* luciferase reporter plasmid pRL-TK (10 ng/well). Next, 24 h posttransfection, cells were stimulated with IFN-α (1,000 U/mL) for 12 h. Cell lysates were then collected to measure luciferase activity using the Dual-Luciferase reporter assay system (Promega, Madison, WI, USA) according to the manufacturer’s instructions and for immunoblotting analysis. Relative luciferase activity was normalized by the ratio of firefly luciferase activity to *Renilla* luciferase activity.

### RNA extraction and qRT-PCR.

Total RNA was extracted using TRIzol reagent (Thermo Fisher Scientific, Waltham, MA, USA) according to the manufacturer’s protocol. cDNA was synthesized using HiScript II Q RT SuperMix for quantitative PCR (qPCR) (plus genomic DNA [gDNA] wiper) (Vazyme Biotech Co., Ltd., Nanjing, China). Quantitative real-time PCR (qRT-PCR) was performed using MonAmp SYBR green qPCR mix (Monad Biotech Co., Ltd., Wuhan, China) on a QuantStudio 3 real-time PCR system (Thermo Fisher Scientific) following the manufacturer’s instructions. The abundance of individual mRNA transcripts in each sample was assayed in triplicate and normalized to the GAPDH mRNA level using the 2^–ΔΔ^*^CT^* method. The primers used for qRT-PCR are listed in Table 1.

**TABLE 1:** Sequences of primers used for qRT-PCR

Primers	Sequence (5′ to 3′)
Human ISG15-forward	GGGACCTGACGGTGAAGATG
Human ISG15-reverse	CGCCGATCTTCTGGGTGAT
Human ISG54-forward	CACCTCTGGACTGGCAATAGC
Human ISG54-reverse	GTCAGGATTCAGCCGAATGG
Human ISG56-forward	GCTTTCAAATCCCTTCCGCTAT
Human ISG56-reverse	GCCTTGGCCCGTTCATAAT
Human OAS1-forward	CGTGTTTCCGCATGCAAATC
Human OAS1-reverse	GCGAACTCAGTACGAAGCTG
Human IRF9-forward	GCCCTACAAGGTGTATCAGTTG
Human IRF9-reverse	TGCTGTCGCTTTGATGGTACT
Human GAPDH-forward	TCATGACCACAGTCCATGCC
Human GAPDH-reverse	GGATGACCTTGCCCACAGCC
Porcine ISG15-forward	CCTGTTGATGGTGCAAAGCT
Porcine ISG15-reverse	TGCACATAGGCTTGAGGTCA
Porcine ISG54-forward	CTGGCAAAGAGCCCTAAGGA
Porcine ISG54-reverse	CTCAGAGGGTCAATGGAATTCC
Porcine ISG56-forward	AAATGAATGAAGCCCTGGAGTATT
Porcine ISG56-reverse	AGGGATCAAGTCCCACAGATTTT
Porcine OAS1-forward	AAGCATCAGAAGCTTTGCATCTT
Porcine OAS1-reverse	CAGGCCTGGGTTTCTTGAGTT
Porcine GAPDH-forward	ACATGGCCTCCAAGGAGTAAGA
Porcine GAPDH-reverse	GATCGAGTTGGGGCTGTGACT

### Immunoblotting and co-IP analyses.

Cells were lysed using cell lysis buffer for Western blotting and IP (Beyotime, Shanghai, China) supplemented with protease/phosphatase inhibitor cocktail (Cell Signaling Technology) and centrifuged at 15,000 × *g* for 15 min at 4°C to remove insoluble cell debris. Protein concentrations in the supernatants were measured using a bicinchoninic acid (BCA) protein assay kit (Biosharp, Anhui, China). For immunoblotting experiments, equal amounts of protein were separated by SDS-PAGE and transferred to polyvinylidene difluoride membranes (Millipore, Darmstadt, Germany). The membranes were blocked with 5% bovine serum albumin in Tris-buffered saline with 0.05% Tween 20 (TBST) for 2 h and subsequently incubated with specific primary antibodies overnight at 4°C. The membranes were then probed with the corresponding secondary antibody for 1 h and finally visualized using the ChemiDoc XRS+ imaging system (Bio-Rad Laboratories, Hercules, CA, USA). Band densitometry was analyzed using Image Lab software version 6.0.0 (Bio-Rad Laboratories) and normalized to control values. For the coimmunoprecipitation (co-IP) experiments, the clarified cell lysates were incubated with anti-HA magnetic beads (Bimake, Houston, TX, USA), anti-Flag magnetic beads (Bimake), or protein A/G magnetic beads (Bimake) precoated with anti-IRF9 antibody at 4°C overnight with gentle rotation. After five washes with phosphate-buffered saline containing 0.5% Tween 20 (PBST) according to the manufacturer’s instructions, the immunoprecipitates were resuspended in 1× SDS loading buffer and boiled for 5 min. The samples were then subjected to immunoblotting analysis using the indicated antibodies.

### Confocal immunofluorescence staining.

Cells seeded in 35-mm glass-bottom cell culture dishes (Biosharp) were fixed with 4% paraformaldehyde for 30 min, permeabilized with 1% Triton X-100 in PBS for 20 min, and then blocked with 5% bovine serum albumin in PBS for 1 h at 37°C. The cells were subsequently incubated with the appropriate primary antibodies diluted in blocking solution at 4°C overnight and stained with Alexa Fluor 488-conjugated goat anti-rabbit IgG, Cy3-conjugated goat anti-mouse IgG, or Alexa Fluor 594-conjugated goat anti-rat IgG secondary antibody for 1 h at 37°C. After that, nuclei were stained with DAPI (Beyotime) for 5 min. The cells were finally mounted using an antifade mounting medium (Beyotime) and visualized using an LSM 880 confocal microscope (Carl Zeiss AG, Oberkochen, Germany).

### Subcellular fractionation.

HEK-293T cells were seeded in 6-well plates and transfected with the indicated expression plasmids or empty vector control. Then, 24 h posttransfection, cells were stimulated with IFN-α (1,000 U/mL) for 4 h. The nuclear and cytoplasmic fractions were extracted using a nuclear and cytoplasmic protein extraction kit (Beyotime) following the manufacturer’s instructions and subjected to immunoblotting analysis.

### Generation of *ATG5* knockout cell lines.

The single guide RNA (sgRNA) sequences targeting the human *ATG5*, sgRNA1 (5′-CATCAAGTTCAGCTCTTCCT-3′) and sgRNA2 (5′-AAATGTACTGTGATGTTCCA-3′) were predicted using the online CRISPR/Cas9 design tool (http://crispr.cos.uni-heidelberg.de) and individually cloned into an all-in-one pYSY-SpCas9-sgRNA-EGFP plasmid (YSY Biotech Co., Ltd., Nanjing, China). The recombinant sgRNA expression plasmids were cotransfected into HEK-293T cells for 24 h. Enhanced green fluorescent protein (EGFP)-positive cells were sorted by flow cytometry using the S3e cell sorter (Bio-Rad Laboratories), and the sorted cells were then seeded into 96-well plates using a limiting dilution method. Positive single-cell clones were validated by DNA sequencing and immunoblotting analyses.

### Generation of ASFV pI215L polyclonal antibody.

The complete ORF *I215L*, lacking the stop codon, was cloned into the pET-30a(+) vector, and the accuracy of the inserts was verified by DNA sequencing. The confirmed recombinant plasmids were then transformed into Escherichia coli strain BL21(DE3) (TransGen Biotech Co., Ltd., Beijing, China) and grown in Luria-Bertani (LB) medium supplemented with 50 μg/mL kanamycin at 37°C. Once the optical density at 600 nm (OD_600_) value reached 0.6, protein expression was induced by adding 1 mM isopropyl-β-d-1-thiogalactopyranoside (IPTG) for an additional 5 h at 37°C. Subsequent purification procedures were performed as described previously (38). Purified His-tagged ASFV pI215L (4 mg) was then used to prepare the anti-pI215L mouse polyclonal antibody by the Laboratory Animal Center, Wuhan Institute of Virology, Chinese Academy of Sciences.

### Ethics statement.

All experiments with live African swine fever viruses were conducted in the animal biosafety level 3 (ABSL-3) laboratory at Huazhong Agricultural University, approved by the Ministry of Agriculture and Rural Affairs and China National Accreditation Service for Conformity Assessment (CNAS).

### Statistical analysis.

Data are expressed as the mean ± standard error of the mean (mean ± SEM) from at least three replicates. Statistical significance of the differences between groups was analyzed using Student’s *t* test or one-way analysis of variance (ANOVA) using Prism version 7.00 (GraphPad Software, Inc., San Diego, CA, USA). A *P* value of <0.05 (*) was considered significant, and *P* values of <0.01 (**) or <0.001 (***) were considered extremely significant.
